# *catena*-Poly[4,4′-bipyridinium [[diaqua­disulfatocadmium(II)]-μ-4,4′-bipyridine-κ^2^ *N*:*N*′] dihydrate]

**DOI:** 10.1107/S1600536809051502

**Published:** 2009-12-12

**Authors:** Wei Xu, Bi-Ju Huang, Ling-Feng Qiu

**Affiliations:** a State Key Laboratory Base of Novel Functional Materials & Preparation Science, Center of Applied Solid State Chemistry Research, Ningbo University, Ningbo 315211, People’s Republic of China

## Abstract

The title compound, {(C_10_H_10_N_2_)[Cd(SO_4_)_2_(C_10_H_8_N_2_)(H_2_O)_2_]·2H_2_O}_*n*_, consists of anionic chains of the Cd complex, diprotonated 4,4′-bipyridinium cations and uncoordinated water mol­ecules. In the anionic chain, the Cd atom lies on a center of inversion in an octa­hedral geometry. The midpoint of the coordinated bipyridine also resides on a center of inversion, as does the non-coordinated bipyridinium counterion. O—H⋯O and N—H⋯O hydrogen bonding inter­actions and π–π stacking inter­actions in the structure are responsible for the supra­molecular assembly.

## Related literature

For background to the structures, topologies and potential applications of metal-organic frameworks, see: Batten & Robson (1998 ▶). For the use of 4,4′-bipyridine (bpy) in the construction of supra­molecular architectures, see: Biradha *et al.* (2006 ▶). For the isostructural complex {(H_2_bpy)[Mn(SO_4_)_2_(bpy)(H_2_O)_2_]·2H_2_O}_*n*_, see: Fan & Zhu (2005 ▶).
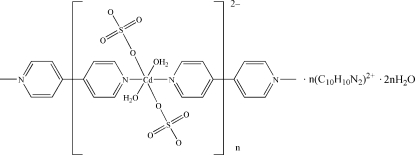


## Experimental

### 

#### Crystal data

(C_10_H_10_N_2_)[Cd(SO_4_)_2_(C_10_H_8_N_2_)(H_2_O)_2_]·2H_2_O
*M* *_r_* = 690.97
Triclinic, 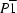

*a* = 7.0150 (14) Å
*b* = 9.4166 (19) Å
*c* = 10.020 (2) Å
α = 74.69 (3)°
β = 88.95 (3)°
γ = 77.89 (3)°
*V* = 623.7 (2) Å^3^
*Z* = 1
Mo *K*α radiation
μ = 1.12 mm^−1^
*T* = 295 K
0.25 × 0.23 × 0.17 mm

#### Data collection

Rigaku R-AXIS RAPID diffractometer
Absorption correction: multi-scan (*ABSCOR*; Higashi, 1995 ▶) *T*_min_ = 0.760, *T*_max_ = 0.830
6106 measured reflections
2797 independent reflections
2572 reflections with *I* > 2σ(*I*)
*R*_int_ = 0.028

#### Refinement

*R*[*F* ^2^ > 2σ(*F* ^2^)] = 0.023
*wR*(*F* ^2^) = 0.058
*S* = 1.06
2797 reflections
198 parameters
H atoms treated by a mixture of independent and constrained refinement
Δρ_max_ = 0.61 e Å^−3^
Δρ_min_ = −0.35 e Å^−3^

### 

Data collection: *RAPID-AUTO* (Rigaku, 1998 ▶); cell refinement: *RAPID-AUTO*; data reduction: *RAPID-AUTO*; program(s) used to solve structure: *SHELXS97* (Sheldrick, 2008 ▶); program(s) used to refine structure: *SHELXL97* (Sheldrick, 2008 ▶); molecular graphics: *SHELXL97*; software used to prepare material for publication: *SHELXL97*.

## Supplementary Material

Crystal structure: contains datablocks global, I. DOI: 10.1107/S1600536809051502/om2300sup1.cif

Structure factors: contains datablocks I. DOI: 10.1107/S1600536809051502/om2300Isup2.hkl

Additional supplementary materials:  crystallographic information; 3D view; checkCIF report

## Figures and Tables

**Table 1:** Hydrogen-bond geometry (Å, °)

*D*—H⋯*A*	*D*—H	H⋯*A*	*D*⋯*A*	*D*—H⋯*A*
O1—H1*A*⋯O4^i^	0.76 (4)	2.11 (4)	2.797 (2)	150
O1—H1*B*⋯O5^ii^	0.84 (3)	1.93 (3)	2.765 (2)	177
O6—H6*A*⋯O4	0.83 (3)	2.14 (3)	2.955 (3)	165
O6—H6*B*⋯O5^iii^	0.87 (4)	2.04 (4)	2.788 (6)	144
N2—H2*A*⋯O3^iv^	0.79 (3)	1.82 (3)	2.602 (6)	170

Symmetry codes: (i) 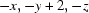; (ii) 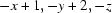; (iii) 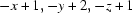; (iv) 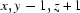.

**Fig. 1.**
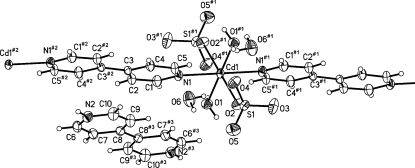
View of the title compound with displacement ellipsoids drawn at the 40% probability level. [Symmetry code: #1 = -x + 1, -y + 2, -z + 1; #2 = -x, -y + 1, -z + 1; #3 = -x + 1, -y + 1, -z + 1]

**Fig. 2.**
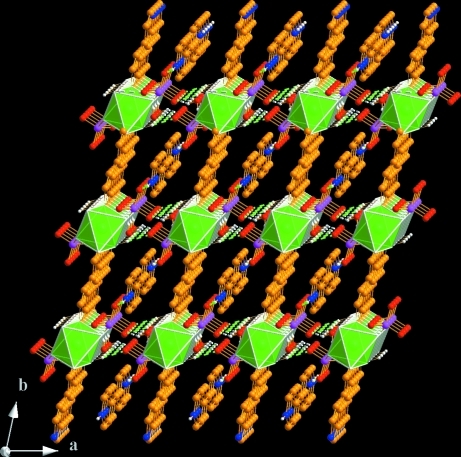
A perspective view of the crystal structure, with hydrogen bonds shown as dashed lines. H atoms not involved in hydrogen bonds have been omitted for clarity.

## supplementary crystallographic information

### Comment

Over the past few decades, much attention has been devoted to the research of novel materials based on metal-organic frameworks (MOFs), motivated by their intriguing structures, new topologies, and potential applications (Batten & Robson, 1998). Since the onset, 4,4'-bipyridine (bpy) has been widely used to construct supramolecular architectures, for it has two potential binding sites which are arranged in a divergent (*exo*) fashion and has a rigid structure which will help in the predictability of network geometries (Biradha, *et al.*, 2006). In the present contribution, we report a new cadmium complex, {(H_2_bpy)[Cd(SO_4_)_2_(bpy)(H_2_O)_2_].2H_2_O}_n_, which is isostructural with the previously reported complex {(H_2_bpy)[Mn(SO_4_)_2_(bpy)(H_2_O)_2_].2H_2_O}_n_ (Fan & Zhu, 2005).

As shown in Fig. 1, the structure consists of {[Cd(SO_4_)_2_(bpy)(H_2_O)_2_)]^2-^}_n_ complex anionic chains, 4,4'-bipyridinium dications and hydrate molecules. The unique Cd atom is coordinated in a slightly distorted octahdedral environment by two N atoms from two bridging 4,4'-bipyridine ligands, two O atoms from two sulfate ligands and two O atoms from two water ligands with Cd—O = 2.282 (1) Å, 2.332 (2) Å and Cd—N = 2.356 (2) Å. The Cd ions are bridged by bpy ligands to give linear –Cd-bpy-Cd- chains, in which the neighbouring cadmium ions are seperated by 11.80 Å. Each sulfate anion acts as a monodentate ligand, and through intra- and intermolecular hydrogen bond interactions with coordinating H_2_O ligands, hydrate molecules and 4,4'-bipyridinium dications to form the three-dimensional network (*d*(O···O) = 2.765–2.955 Å and d(O···N) = 2.602 Å) (Fig.2 and Table 1). The 4,4'-bipyridinium dications with the neighboring bpy in the one-dimensional chains form π-π stacking interactions with a distance of about 3.42 Å.

### Experimental

0.156 g (1 mmol) 4,4'-bipyridine and 0.151 g (1 mmol) DL-mercaptosuccinic acid were dissolved with stirring in aqueous methanol (20 ml, 1:1 *v*/*v*). A total of 0.256 g (1 mmol) CdSO_4_.8/3H_2_O was added to the above solution to obtain a cloudy solution (pH = 3.74), which was filtered. The resulting colorless filtrate was maintained at room temperature and afforded colorless crystals two week later by slow evaporation (yield 18% based on the initial CdSO_4_.8/3H_2_O input).

### Refinement

H atoms bonded to C atoms were placed in geometrically calculated positions and were refined using a riding model, with *U*_iso_(H) = 1.2 *U*_eq_(C). H atoms attached to O atoms were found in a difference Fourier synthesis and refined freely.

### Crystal data

**Table:**

(C_10_H_10_N_2_)[Cd(SO_4_)_2_(C_10_H_8_N_2_)(H_2_O)_2_]·2H_2_O	*Z* = 1
*M**_r_* = 690.97	*F*(000) = 350
Triclinic, *P*1	*D*_x_ = 1.840 Mg m^−^^3^
Hall symbol: -P 1	Mo *K*α radiation, λ = 0.71073 Å
*a* = 7.0150 (14) Å	Cell parameters from 5820 reflections
*b* = 9.4166 (19) Å	θ = 3.4–27.5°
*c* = 10.020 (2) Å	µ = 1.12 mm^−^^1^
α = 74.69 (3)°	*T* = 295 K
β = 88.95 (3)°	Block, light-yellow
γ = 77.89 (3)°	0.25 × 0.23 × 0.17 mm
*V* = 623.7 (2) Å^3^	

### Data collection

**Table:**

Rigaku R-AXIS RAPID diffractometer	2797 independent reflections
Radiation source: fine-focus sealed tube	2572 reflections with *I* > 2σ(*I*)
graphite	*R*_int_ = 0.028
ω scans	θ_max_ = 27.5°, θ_min_ = 3.4°
Absorption correction: multi-scan (*ABSCOR*; Higashi, 1995)	*h* = −8→9
*T*_min_ = 0.760, *T*_max_ = 0.830	*k* = −12→12
6106 measured reflections	*l* = −12→12

### Refinement

**Table:**

Refinement on *F*^2^	Primary atom site location: structure-invariant direct methods
Least-squares matrix: full	Secondary atom site location: difference Fourier map
*R*[*F*^2^ > 2σ(*F*^2^)] = 0.023	Hydrogen site location: inferred from neighbouring sites
*wR*(*F*^2^) = 0.058	H atoms treated by a mixture of independent and constrained refinement
*S* = 1.06	*w* = 1/[σ^2^(*F*_o_^2^) + (0.0368*P*)^2^] where *P* = (*F*_o_^2^ + 2*F*_c_^2^)/3
2797 reflections	(Δ/σ)_max_ < 0.001
198 parameters	Δρ_max_ = 0.61 e Å^−^^3^
0 restraints	Δρ_min_ = −0.35 e Å^−^^3^

### Special details

**Table:**

Geometry. All esds (except the esd in the dihedral angle between two l.s. planes) are estimated using the full covariance matrix. The cell esds are taken into account individually in the estimation of esds in distances, angles and torsion angles; correlations between esds in cell parameters are only used when they are defined by crystal symmetry. An approximate (isotropic) treatment of cell esds is used for estimating esds involving l.s. planes.
Refinement. Refinement of F^2^ against ALL reflections. The weighted R-factor wR and goodness of fit S are based on F^2^, conventional R-factors R are based on F, with F set to zero for negative F^2^. The threshold expression of F^2^ > 2sigma(F^2^) is used only for calculating R-factors(gt) etc. and is not relevant to the choice of reflections for refinement. R-factors based on F^2^ are statistically about twice as large as those based on F, and R- factors based on ALL data will be even larger.

### Fractional atomic coordinates and isotropic or equivalent isotropic displacement parameters (Å^2^)

**Table:**

	*x*	*y*	*z*	*U*_iso_*/*U*_eq_	
Cd1	0.0000	1.0000	0.0000	0.01975 (7)	
S1	0.29197 (7)	1.15768 (5)	0.17257 (4)	0.02264 (11)	
O1	0.1945 (2)	0.84005 (18)	−0.11454 (17)	0.0314 (3)	
H1A	0.136 (6)	0.833 (4)	−0.175 (4)	0.088 (14)*	
H1B	0.290 (4)	0.868 (3)	−0.157 (3)	0.043 (8)*	
O2	0.2561 (2)	1.08582 (18)	0.06347 (15)	0.0317 (3)	
O3	0.3055 (3)	1.31472 (19)	0.10627 (17)	0.0436 (4)	
O4	0.1344 (2)	1.15595 (19)	0.27022 (15)	0.0368 (4)	
O5	0.4799 (2)	1.0756 (2)	0.24417 (17)	0.0450 (4)	
N1	0.0296 (2)	0.79779 (18)	0.20108 (16)	0.0239 (3)	
C1	0.0538 (3)	0.6552 (2)	0.19390 (19)	0.0256 (4)	
H1	0.0766	0.6356	0.1081	0.031*	
C2	0.0466 (3)	0.5356 (2)	0.30742 (19)	0.0243 (4)	
H2	0.0672	0.4381	0.2975	0.029*	
C3	0.0080 (3)	0.56194 (19)	0.43746 (18)	0.0196 (3)	
C4	−0.0128 (3)	0.7102 (2)	0.44466 (19)	0.0252 (4)	
H4	−0.0347	0.7334	0.5290	0.030*	
C5	−0.0006 (3)	0.8224 (2)	0.3263 (2)	0.0262 (4)	
H5	−0.0140	0.9204	0.3338	0.031*	
N2	0.4126 (3)	0.3731 (2)	0.85093 (18)	0.0298 (4)	
H2A	0.385 (4)	0.344 (3)	0.929 (3)	0.036 (7)*	
C6	0.4633 (3)	0.2724 (2)	0.7777 (2)	0.0308 (4)	
H6	0.4744	0.1704	0.8203	0.037*	
C7	0.4990 (3)	0.3192 (2)	0.6395 (2)	0.0284 (4)	
H7	0.5344	0.2489	0.5886	0.034*	
C8	0.4823 (3)	0.4724 (2)	0.57526 (19)	0.0237 (4)	
C9	0.4306 (3)	0.5733 (2)	0.6567 (2)	0.0331 (5)	
H9	0.4192	0.6761	0.6178	0.040*	
C10	0.3968 (3)	0.5197 (3)	0.7943 (2)	0.0346 (5)	
H10	0.3624	0.5867	0.8486	0.041*	
O6	0.2828 (3)	0.9313 (2)	0.5331 (2)	0.0477 (4)	
H6A	0.257 (5)	1.003 (4)	0.462 (3)	0.057 (9)*	
H6B	0.376 (5)	0.956 (4)	0.572 (4)	0.064 (10)*	

### Atomic displacement parameters (Å^2^)

**Table:**

	*U*^11^	*U*^22^	*U*^33^	*U*^12^	*U*^13^	*U*^23^
Cd1	0.02501 (11)	0.01778 (10)	0.01586 (10)	−0.00645 (7)	−0.00012 (7)	−0.00191 (7)
S1	0.0271 (2)	0.0267 (3)	0.0168 (2)	−0.01104 (19)	0.00086 (18)	−0.00635 (18)
O1	0.0293 (8)	0.0344 (8)	0.0308 (8)	−0.0052 (6)	0.0055 (7)	−0.0106 (6)
O2	0.0315 (7)	0.0432 (9)	0.0301 (8)	−0.0156 (6)	0.0030 (6)	−0.0206 (7)
O3	0.0716 (11)	0.0319 (9)	0.0338 (8)	−0.0240 (8)	0.0179 (8)	−0.0110 (7)
O4	0.0407 (8)	0.0481 (10)	0.0286 (8)	−0.0195 (7)	0.0121 (7)	−0.0154 (7)
O5	0.0375 (8)	0.0620 (12)	0.0330 (9)	−0.0084 (8)	−0.0092 (7)	−0.0093 (8)
N1	0.0291 (8)	0.0226 (8)	0.0183 (7)	−0.0080 (6)	−0.0015 (7)	−0.0005 (6)
C1	0.0338 (10)	0.0258 (10)	0.0164 (8)	−0.0068 (8)	0.0011 (8)	−0.0037 (7)
C2	0.0332 (10)	0.0187 (9)	0.0208 (9)	−0.0060 (7)	0.0016 (8)	−0.0048 (7)
C3	0.0207 (8)	0.0179 (9)	0.0177 (8)	−0.0035 (7)	−0.0011 (7)	−0.0010 (7)
C4	0.0368 (10)	0.0207 (9)	0.0179 (8)	−0.0064 (8)	0.0009 (8)	−0.0043 (7)
C5	0.0383 (10)	0.0175 (9)	0.0214 (9)	−0.0072 (8)	−0.0010 (8)	−0.0014 (7)
N2	0.0336 (9)	0.0325 (10)	0.0217 (9)	−0.0090 (7)	0.0035 (8)	−0.0031 (7)
C6	0.0348 (10)	0.0243 (10)	0.0290 (10)	−0.0048 (8)	0.0015 (9)	−0.0007 (8)
C7	0.0317 (10)	0.0235 (10)	0.0284 (10)	−0.0033 (8)	0.0025 (8)	−0.0062 (8)
C8	0.0219 (8)	0.0244 (10)	0.0233 (10)	−0.0054 (7)	−0.0015 (7)	−0.0033 (7)
C9	0.0461 (12)	0.0251 (11)	0.0285 (10)	−0.0098 (9)	0.0034 (9)	−0.0059 (8)
C10	0.0466 (12)	0.0317 (11)	0.0279 (10)	−0.0106 (9)	0.0039 (10)	−0.0107 (8)
O6	0.0668 (12)	0.0383 (10)	0.0360 (10)	−0.0107 (9)	−0.0098 (9)	−0.0058 (8)

### Geometric parameters (Å, °)

**Table:**

Cd1—O2	2.2821 (14)	C3—C3^ii^	1.491 (3)
Cd1—O2^i^	2.2821 (14)	C4—C5	1.379 (3)
Cd1—O1	2.3324 (17)	C4—H4	0.9300
Cd1—O1^i^	2.3324 (17)	C5—H5	0.9300
Cd1—N1	2.3562 (18)	N2—C10	1.328 (3)
Cd1—N1^i^	2.3562 (18)	N2—C6	1.334 (3)
S1—O4	1.4625 (16)	N2—H2A	0.80 (3)
S1—O5	1.4713 (17)	C6—C7	1.373 (3)
S1—O3	1.4747 (17)	C6—H6	0.9300
S1—O2	1.4793 (14)	C7—C8	1.397 (3)
O1—H1A	0.77 (4)	C7—H7	0.9300
O1—H1B	0.84 (3)	C8—C9	1.397 (3)
N1—C1	1.338 (2)	C8—C8^iii^	1.494 (4)
N1—C5	1.341 (2)	C9—C10	1.373 (3)
C1—C2	1.382 (3)	C9—H9	0.9300
C1—H1	0.9300	C10—H10	0.9300
C2—C3	1.401 (3)	O6—H6A	0.84 (3)
C2—H2	0.9300	O6—H6B	0.87 (3)
C3—C4	1.394 (3)		
			
O2—Cd1—O2^i^	180.0	C1—C2—C3	119.64 (17)
O2—Cd1—O1	93.80 (6)	C1—C2—H2	120.2
O2^i^—Cd1—O1	86.20 (6)	C3—C2—H2	120.2
O2—Cd1—O1^i^	86.20 (6)	C4—C3—C2	116.60 (16)
O2^i^—Cd1—O1^i^	93.80 (6)	C4—C3—C3^ii^	121.4 (2)
O1—Cd1—O1^i^	180.0	C2—C3—C3^ii^	122.0 (2)
O2—Cd1—N1	94.14 (6)	C5—C4—C3	119.85 (17)
O2^i^—Cd1—N1	85.86 (6)	C5—C4—H4	120.1
O1—Cd1—N1	89.48 (6)	C3—C4—H4	120.1
O1^i^—Cd1—N1	90.52 (6)	N1—C5—C4	123.46 (18)
O2—Cd1—N1^i^	85.86 (6)	N1—C5—H5	118.3
O2^i^—Cd1—N1^i^	94.14 (6)	C4—C5—H5	118.3
O1—Cd1—N1^i^	90.52 (6)	C10—N2—C6	121.81 (19)
O1^i^—Cd1—N1^i^	89.48 (6)	C10—N2—H2A	119.5 (19)
N1—Cd1—N1^i^	180.00 (7)	C6—N2—H2A	118.6 (19)
O4—S1—O5	110.73 (10)	N2—C6—C7	120.1 (2)
O4—S1—O3	109.56 (11)	N2—C6—H6	119.9
O5—S1—O3	108.83 (11)	C7—C6—H6	119.9
O4—S1—O2	110.99 (9)	C6—C7—C8	120.0 (2)
O5—S1—O2	108.04 (10)	C6—C7—H7	120.0
O3—S1—O2	108.62 (9)	C8—C7—H7	120.0
Cd1—O1—H1A	110 (3)	C7—C8—C9	117.66 (19)
Cd1—O1—H1B	118.7 (19)	C7—C8—C8^iii^	121.5 (2)
H1A—O1—H1B	100 (3)	C9—C8—C8^iii^	120.8 (2)
S1—O2—Cd1	134.77 (9)	C10—C9—C8	119.6 (2)
C1—N1—C5	117.01 (16)	C10—C9—H9	120.2
C1—N1—Cd1	121.51 (12)	C8—C9—H9	120.2
C5—N1—Cd1	121.00 (13)	N2—C10—C9	120.7 (2)
N1—C1—C2	123.39 (17)	N2—C10—H10	119.6
N1—C1—H1	118.3	C9—C10—H10	119.6
C2—C1—H1	118.3	H6A—O6—H6B	101 (3)

Symmetry codes: (i) −*x*, −*y*+2, −*z*; (ii) −*x*, −*y*+1, −*z*+1; (iii) −*x*+1, −*y*+1, −*z*+1.

### Hydrogen-bond geometry (Å, °)

**Table:**

*D*—H···*A*	*D*—H	H···*A*	*D*···*A*	*D*—H···*A*
O1—H1A···O4^i^	0.76 (4)	2.11 (4)	2.797 (2)	150
O1—H1B···O5^iv^	0.84 (3)	1.93 (3)	2.765 (2)	177
O6—H6A···O4	0.83 (3)	2.14 (3)	2.955 (3)	165
O6—H6B···O5^v^	0.87 (4)	2.04 (4)	2.788 (6)	144
N2—H2A···O3^vi^	0.79 (3)	1.82 (3)	2.602 (6)	170

Symmetry codes: (i) −*x*, −*y*+2, −*z*; (iv) −*x*+1, −*y*+2, −*z*;  (v) −*x*+1, −*y*+2, −*z*+1; (vi) *x*, *y*−1, *z*+1.
